# Reducing the Risk of Birth Defects Associated with Maternal Influenza: Insights from a Hungarian Case—Control Study

**DOI:** 10.3390/jcm12216934

**Published:** 2023-11-05

**Authors:** Ákos Mátrai, Brigitta Teutsch, Boglárka Pethő, András D. Kaposi, Péter Hegyi, Nándor Ács

**Affiliations:** 1 Department of Obstetrics and Gynecology, Semmelweis University, 1082 Budapest, Hungary; matraiakos@gmail.com (Á.M.); pethobogi@gmail.com (B.P.); 2 Faculty of Health Sciences, University of Pécs, 7621 Pécs, Hungary; 3 Centre for Translational Medicine, Semmelweis University, 1085 Budapest, Hungary; teutschbrigitta@gmail.com (B.T.); hegyi2009@gmail.com (P.H.); 4 Institute for Translational Medicine, Medical School, University of Pécs, 7623 Pécs, Hungary; 5 Department of Biophysics and Radiation Biology, Semmelweis University School of Medicine, 1094 Budapest, Hungary; kaposi.andras@gmail.com; 6 Institute of Pancreatic Diseases, Semmelweis University, 1083 Budapest, Hungary

**Keywords:** influenza, pregnancy, first trimester, non-chromosomal congenital malformations

## Abstract

Influenza viruses can cause several complications during pregnancy. Therefore, we aimed to investigate the effects of influenza on the development of congenital abnormalities (CAs) by analyzing the database of the Hungarian Case–Control Surveillance of Congenital Abnormalities (HCCSCA). In our multicenter, case–control, population-based study, we processed clinician-reported outcomes and diagnoses collected in the HCCSCA. The case group included newborns with different non-chromosomal birth defects, while the controls were newborns without CAs. Maternal influenza, as a risk factor for CAs, was analyzed by using a logistic regression model and odds ratios with 95% confidence intervals (CIs). Our results showed that maternal influenza in the first trimester was associated with increased odds of developing non-chromosomal CAs (OR: 1.41, CI: 1.28–1.55). There were increased odds of neural tube defects (OR: 2.22, CI: 1.78–2.76), orofacial clefts (OR: 2.28, CI: 1.87–2.78), and congenital heart defects (OR: 1.28, CI: 1.10–1.49) after influenza infection. In all cases, we found a protective effect of folic acid supplementation in the first trimester. In summary, the odds of non-chromosomal birth defects are higher after maternal influenza in the first trimester, and folic acid or pregnancy vitamin supplementation and antipyretic therapy may reduce the effect of maternal influenza during the first trimester.

## 1. Introduction

Since 2020, the COVID-19 pandemic has again highlighted the risk of viral infections during pregnancy. Moreover, compared to COVID-19, the incidence of influenza is much higher in pregnant women [1].

Influenza is a seasonal viral infection that affects the respiratory system and occurs in epidemic outbreaks during the winter. It is usually spread through droplets when someone coughs or sneezes. The symptoms include a sudden onset of fever, chills, headache, muscle aches, sore throat, fatigue, and a runny nose. It is caused by influenza viruses A, B, or C; the incubation period is about two days. Recovery usually lasts 3–7 days for healthy individuals, but older adults, children, pregnant women, and people with chronic diseases are at increased risk of complications. Therefore, it is important to follow precautions, such as hand washing and wearing a mask, to reduce its spread and protect our health [2].

The first 12 to 13 weeks of pregnancy are when organogenesis occurs. The incidence of congenital abnormalities (CAs) is 3–5% worldwide. Congenital abnormalities are considered the leading causes of infant mortality. Influenza can also occur during pregnancy [3]. During this critical time frame, in the first trimester, it can have a deleterious effect on the developing embryo, leading to various birth defects (BDs) [4]. The meta-analysis published by our working group last year also revealed associations between maternal influenza and the development of several malformations [5].

The aim of the present study was to estimate the odds of birth defects in the offspring of mothers with influenza during the first trimester in the Hungarian population. We hypothesized that influenza in the first trimester of pregnancy increases the likelihood of developing non-chromosomal CAs.

## 2. Materials and Methods

This study is reported according to the recommendations of the STROBE guidelines (Supplementary Table S1) [6].

### 2.1. Study Design and Setting

In our case–control study, we investigated the effects of maternal influenza during the first trimester of pregnancy on the development of non-chromosomal birth defects in the Hungarian population. The cases and controls were enrolled in the database of the Hungarian Case–Control Surveillance of Congenital Abnormalities (HCCSCA) between 1980 and 2009.

### 2.2. Data Collection

The HCCSCA was established in 1980 [7], and the cases were recorded from the Hungarian Congenital Abnormality Registry (HCAR). This dataset contains details on 90,000 pregnancies. The data collection was changed in 1997, slightly modifying the structure of the dataset [8]. In a previous project, data collected in the HCCSCA between 1980 and 2009 were combined into an authenticated, unified database [8].

The HCAR was established in 1962 as the first national-based registry of CAs in the world [9]. Physicians were required to report patients with CAs to the HCAR from birth to the end of the first postnatal year. Given that almost all births in Hungary take place in inpatient obstetric wards, all deliveries were reported by obstetricians and pediatricians [9]. After 1984, prenatal diagnosis centers were also required to report malformed fetuses diagnosed prenatally to the HCAR. In each case, two designated geneticists from HCAR examined the affected newborns. The diagnosis of CA was confirmed with a physical examination, and then, they were divided into subgroups.

### 2.3. Participants

Three criteria were used to determine which cases with CAs from the HCAR were included in the HCCSCA: (1) cases reported within three months of birth or elective termination of pregnancy and (2) cases that did not have dislocation of the hip, congenital inguinal hernia, or large hemangiomas with included, and (3) cases with a co-occurrence of chromosomal abnormalities were excluded. The origin of CA syndromes is periconceptional. The HCCSCA was used to detect teratogenic or fetotoxic effects during pregnancy.

The controls were specified as newborn infants without any CAs. They were matched to every case according to sex, birth week, and district of residence of the parents. These controls were selected from the Hungarian National Birth Registry of the Central Statistical Office based on the HCCSCA case lists for each quarter of the year. If the controls were twins, only one was randomly selected for the HCCSCA [8].

### 2.4. Variables and Data Sources

The following data were collected for each case and control patient in the database: CA(s), gender, birth year/month/day, birth weight, gestational age, area of residence of mother, maternal age, paternal age, birth order, education of mother and father, status and type of employment, marital status of mother, outcomes of previous pregnancies, maternal diseases during pregnancy (by month of pregnancy), medication taken during pregnancy (by month of pregnancy), folic acid and/or pregnancy vitamin supplement taken (by month of pregnancy), smoking habits, and alcohol consumption patterns of mother [8].

The data were collected in three ways: prospective data collection using medical records, retrospective self-reported data about mothers, and supplementary data collection.

Prospective data collection using medical records:

Immediately following the selection of cases, controls, and malformed controls, the mothers received an educational letter and an informed consent form. The aim of this correspondence was to underscore the importance of the undertaking. In addition to other medical records, mothers were asked to submit their prenatal care logbook. Of particular interest were the discharge summaries from their hospital stays, which included information about their offspring’s congenital anomalies in addition to a detailed account of their medical conditions and the medications they received during the studied pregnancy. Within a month, these records were returned. Nearly all expectant mothers in Hungary receive prenatal care; the first appointment occurs between weeks six and twelve of gestation, and there are typically seven further visits after that. During these prenatal care sessions, obstetricians record in the prenatal care logbook any pregnancy difficulties, maternal health concerns, and prescribed medications [8].

Retrospective self-reported data about mothers:

The mothers of the infants with congenital abnormalities, controls, and cases were given a structured, printed questionnaire that included a detailed inventory of all prescribed drugs and medical conditions. Information regarding their marital and employment status, use of prescription drugs and pregnancy supplements, difficulties related to pregnancy, and maternal health concerns during pregnancy were also requested in this questionnaire. Documentation was also carried out regarding prior pregnancies and family histories of congenital abnormalities. Mothers were advised to use the supplied list of medications and medical problems as a memory aid before responding in order to guarantee consistency in their responses [8].

Supplementary data collection:

Local nurses visited the homes of both the patients and the controls after 1996. The mothers received assistance from these nurses in compiling their medical records and filling out the questionnaire. However, a mother who expressed worries about data privacy in 2002 called into question this data harvesting procedure. As a result, when court proceedings started in 2003, the HCCSCA’s operations were briefly put on hold. The organization was only able to restart operations in 2005 [8].

In the HCCSCA, there is no exact record about the source of each and every piece of data; only the information gained is recorded in the database. In 100% of the cases, the data were obtained from the pregnancy logbooks (the availability of medical records was an inclusion criterion). In all, 72.9% of the mothers answered the questionnaires, and 11.7% of the families were visited personally [7].

The analysis used the International Classification of Diseases (ICD)-10 to identify congenital malformations and diseases during the analysis. Thus, the definition of influenza infection was based on reported data and ICD-10 definitions. For most major CAs, the critical period is the first trimester, i.e., the first three months of pregnancy. Therefore, cases were analyzed only if they met two criteria: an influenza infection was reported in the first three gestational months, and the National Center for Epidemiology recorded an influenza epidemic during that period. Gestational age was calculated from the first day of the last menstrual period.

### 2.5. Statistical Methods

Data were extracted from the unified database of the Hungarian Case–Control Surveillance of Congenital Abnormalities into a Microsoft Excel datasheet, stored in data tables, and re-validated before being used for data analysis. Statistical analysis was performed using R statistical software [10]. After the data were described, a logistic regression model was fitted: we used the rate of congenital abnormalities as the response variable and the first-trimester influenza rate as the explanatory variable. The effect was adjusted for the intake of antipyretic drugs, folic acid, and maternal vitamins. There was no interaction term in the final model. Maternal age (continuous variable) and birth order (parity) (2 vs. 1, 3+ vs. 1), as well as job position (skilled/semiskilled worker vs. professional/managerial, unskilled worker/other vs. professional/managerial) as a measure of socioeconomic class, were taken into consideration as confounding factors. The case and control groups had the same distribution of these possible confounding variables. To report our results, we used the prevalence odds ratios, their 95% confidence intervals (CIs), and the outcome probabilities. A *p*-value of less than 0.05 was considered statistically significant.

## 3. Results

### 3.1. Participants

**Figure 1:**
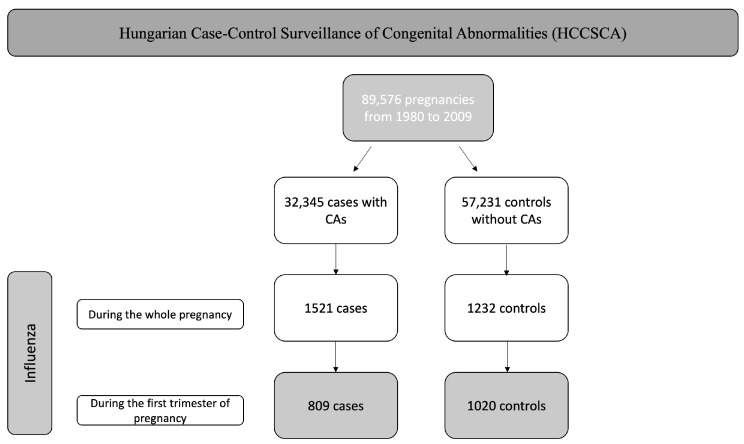
Flowchart of the selection.

In the unified HCCSCA database, we identified 32,345 cases and 57,231 controls, altogether 89,576 pregnancies. Of the 32,345 cases, 809 cases of influenza infection occurred during the first trimester. Of the 57,231 controls, we identified 1020 control cases of influenza during the first trimester. Between 1980 and 2009, there were 3,009,303 live births in Hungary; therefore, our control group represents 1.9% of the total births in the country. These are shown in Figure 1.

### 3.2. Descriptive Data

*Basic Characteristics of Patients Included*

The baseline characteristics of patients included maternal age, folic acid intake, pregnancy vitamin intake, and the use of antipyretic therapy. These are presented in Table 1.

### 3.3. Outcome Data

*Non-Chromosomal Congenital Abnormalities*

We found a significant association between the development of maternal influenza in the first trimester and some non-chromosomal congenital malformations. If influenza was present in the first three months of pregnancy, the odds of the development of non-chromosomal malformations increased almost one and a half times (OR: 1.41, CI: 1.28–1.55; *p* < 0.001).

We found possible associations between influenza during the second and third months of pregnancy and three groups of CAs (neural tube defects, oral clefts, and congenital heart defects) in the analysis of 25 CA groups. These CA groups are among the most frequent ones and have a clinically relevant impact on the life of the newborn. Of the 809 cases, we identified 88 cases of neural tube defects (NTDs), 110 cases of oral clefts, and 190 cases of cardiovascular malformations.

*Neural Tube Defects*

Our findings highlighted a twofold increase in the development of NTDs (OR: 2.22, CI: 1.78–2.76; *p* < 0.001) after influenza infection in the first trimester. We also performed a subgroup analysis for NTDs. The results showing the increased odds of developing spina bifida, hydrocephalus, anencephaly, and encephalocele after maternal influenza are shown in Table 2.

*Oral Clefts*

We found a significant increase in the odds of developing oral clefts after maternal influenza in the first trimester (OR: 2.28, CI: 1.87–2.78; *p* < 0.001). The subgroup analysis found a more than twofold increase in the odds for cleft lip, cleft palate with unilateral cleft lip, and unspecified cleft palate development. These findings are shown in Table 3.

*Congenital Heart Defects*

Overall, our findings also highlighted increased odds of developing congenital heart defects (CHDs) (OR: 1.28, CI: 1.10–1.49; *p* < 0.001). We found significant associations when the subgroups were analyzed. The odds of developing ventricular septal defects increased after maternal influenza in the first trimester (OR: 1.38, CI: 1.09–1.72; *p* = 0.006). The odds for different subcategories are shown in Table 4.

*Effects of the Drugs Used*

After determining the odds ratios after maternal influenza in the first trimester, we examined the association of folic acid use, the use of pregnancy multivitamins, and antipyretic therapy with the development of congenital malformations. We identified minimal odds ratio reductions for NTDs and oral clefts. For heart defects, we found a protective effect of folic acid supplementation in the first trimester, as indicated by the reduced odds ratio, but these findings were not significant. These findings are shown in Table 5.

We also analyzed the use of pregnancy multivitamins and identified a protective effect of multivitamin supplementation on the development of neural tube defects. These findings are shown in Table 6.

Finally, we also examined whether the development of congenital malformations was affected by antipyretics when influenza occurred in the first trimester. We found no association between the use of antipyretics and the occurrence of all types of birth defects. However, in the case of neural tube defects, antipyretics reduced the odds. These findings are shown in Table 7.

## 4. Discussion

The first trimester, i.e., the first 12 weeks of pregnancy, is particularly important in the development of birth defects. Organogenesis takes place during this crucial period, and any environmental impact on the mother can, therefore, adversely affect this developmental process [3]. Estimates place the key period for the majority of CAs between the 35th and 84th post-conceptional days, or the 49th and 98th gestational days measured from the first day of the most recent menstrual cycle. As a result, exposures need to be assessed in the second and third months of pregnancy. A cleft palate’s critical period is the only exception; exposures for the condition are assessed in the third and fourth gestational months. This period is estimated to be between the 70th and 99th postconceptional days or the 84th and 113th gestational days measured from the first day of the last menstrual period [11]. In the current pandemic period, the importance of viral infections has been re-emphasized [1]. However, the emergence of COVID-19 should not be overlooked, nor should the influenza virus, which appears as an epidemic every year. Since the 1980s, numerous studies have investigated the adverse effects of influenza on pregnancy outcomes and the development of birth defects [5,12,13,14,15].

While there has been much research on the connection between influenza illness during the first trimester and congenital abnormalities, this study definitely validates what is already known. With information from 90,000 pregnancies in a homogeneous community, the Hungarian Case–Control Surveillance of Congenital Abnormalities database is unique in the world. The amount of data and the high degree of reliability of the data gathering process have led to high power in the data processing of this well-designed study [8].

The overall findings represented increased odds of developing non-chromosomal birth defects after the first trimester of maternal influenza. In line with our findings, Oster et al. from the United States of America (USA) reported a large case–control study of nearly 6000 patients with the same conclusion for influenza infection in the first trimester [15]. According to the literature and the meta-analysis previously performed by our working group, neural tube defects, oral clefts, and cardiovascular malformations are the three major types of non-chromosomal birth defects, with an increased prevalence after influenza infection [5]. These CA categories are among the most common and affect the newborn’s life in a way that is clinically significant.

Brain, spine, or spinal cord birth malformations are known as neural tube defects. They typically occur in the first month of pregnancy, sometimes even before a woman is aware that she is carrying a child. Reviewing the literature, Saxén et al. [16] and Kurppa et al. [17] investigated the development of neural tube defects and reported the same increased odds of the development of NTDs after maternal influenza in the first trimester. Among the causes of NTDs, maternal factors should also be mentioned, such as obesity, hyperthermia (fever), and diabetes. Valproate intake or low folate intake is also known as the main risk factor for the disease [18]. Therefore, it is essential to raise awareness among pregnant women about folic acid supplementation or prenatal vitamin intake even before becoming pregnant.

Oral cleft screening is part of the ultrasound scan at week 20. These types of birth defects occur in 1.5 out of 1000 live births [19]. Newborns born with these anomalies have feeding difficulties, speech problems, or conductive hearing loss [19]. Our results suggest that maternal influenza in the first trimester is a risk factor for developing these congenital malformations. Saxén et al. [20,21] and Dymanus et al. [22] confirmed the link between maternal influenza and the development of orofacial clefts. A previous study based on our database confirmed that maternal influenza occurring in the first trimester and other lifestyle factors (gender, birthweight, smoking) are risk factors for the development of cleft palate [23]. Among the causes of oral clefts, alcohol consumption, anticonvulsive therapy, or folic acid deficiency must be mentioned [24].

Particular attention should be paid to congenital heart defects. These birth defects are common anomalies and are leading causes of neonatal or infant deaths. After reviewing the literature, we found that many studies [12,13] have investigated the origin of cardiovascular malformations. A previous meta-analysis supports the fact that the risk of congenital heart defects increases in the case of viral infections during early pregnancy [25]. Among the underlying causes confirming our findings, maternal influenza infection [13,14,26], low maternal employment status, pregestational diabetes, maternal clotting disorders, and prescriptions of anticoagulants should be mentioned [15].

Focusing on the prevention of these types of anomalies, there are several studies in the literature confirming our findings. Several studies have concluded that folic acid supplementation reduces the chance of some developmental disorders [18]. The pathomechanism of influenza that leads to the development of congenital malformations is unknown, which is why studies into the effects of antipyretic medications are being conducted. Several studies have raised the possibility that the causative factor is fever and not the infection itself. Therefore, since our database also contains reliable data on the use of antipyretic drugs, the aim of our research was to analyze the potential protective effect of these drugs on the development of congenital anomalies in influenza patients. Antipyretic medication use during influenza was examined in several trials, and the results showed that using these medications decreased the risk of some birth abnormalities [12]. However, our results did not show evidence that antipyretics decreased the risk of all forms of malformations. This effect was limited to what we saw in neural tube abnormalities.

### 4.1. Bias

Despite the long study period, the specific nature of data collection and verification increases the reliability of the data. However, the classification of the International Classification of Diseases (ICD) changed during the study period several times, so the conversion of different ICD categories into each other may have caused minor differences in the outcome groups. As with all case–control studies involving interviews and questionnaires, recall bias reduces the reliability of this study. Recall bias might have played a role in the assessed associations. However, the diagnosis of influenza was only accepted if it was medically recorded. Thus, in this case, recall bias cannot affect the results of this study.

### 4.2. Strengths and Limitations

Among the strengths of our analysis, we must mention the large number of cases and the HCCSCA database, which is unique in the world due to its extensiveness. We can also mention the data collection methods, which contributed to data quality. Among the strengths, it should also be mentioned that the controls were assigned to the cases thanks to very precise matching.

As for the limitations of this analysis, although data collection was performed using three methods, the identification of influenza was determined based on the symptoms and self-reported retrospective data. Thus, due to the measurement of risk factors and case–control studies, the risk of bias was high.

### 4.3. Implications for Practice and Research

The translation of scientific findings into everyday patient care is decisive in medicine today; therefore, we should try to prevent influenza during pregnancy [27]. We must raise awareness among pregnant women about the safe use of vaccination. Awareness-raising campaigns should be organized on the importance of folic acid and its modern counterpart, pregnancy vitamins. In the future, it would be recommended to start a prospective study in which influenza would be identified on the basis of polymerase chain reaction (PCR).

## 5. Conclusions

Our findings suggest that maternal influenza occurring in the first trimester is associated with non-chromosomal birth defects, mainly neural tube defects, oral clefts, and congenital heart defects. Prevention is of significant importance, including vaccination and folic acid/pregnancy vitamin intake.

## Figures and Tables

**Table 1:** Baseline characteristics of patients in the case and control groups.

	First-Trimester Influenza Infection	
Characteristic	Cases (*n* = 809)	Controls (*n* = 1020)
**Maternal age**		
<25	392 (48.45%)	462 (45.29%)
25–29	252 (31.15%)	357 (35.00%)
>29	165 (20.40%)	201 (19.71%)
Mean ± SD	25.61 ± 5.27	25.53 ± 4.72
**Folic acid supplementation**		
Yes	328 (40.54%)	490 (48.04%)
No	481 (59.46%)	530 (51.96%)
**Pregnancy vitamin supplementation**		
Yes	65 (8.03%)	68 (6.67%)
No	744 (91.97%)	952 (93.33%)
**Use of antipyretics**		
Yes	250 (30.90%)	322 (31.57%)
No	559 (69.10%)	698 (68.43%)

**Table 2:** Detailed odds ratios for developing neural tube defects.

	Case Mothers	Matched Controls	Comparison	
Type of CAs	No.	No.	Odds Ratio	95%–CI
Neural tube defects	88 (4.81%)	1954 (2.23%)	2.22	1.78–2.76
Spina bifida	30 (1.64%)	578 (0.66%)	2.52	1.70–3.57
Hydrocephalus	19 (1.04%)	289 (0.33%)	3.18	1.93–4.92
Anencephalia	13 (0.71%)	301 (0.34%)	2.08	1.31–3.48
Encephalocele	10 (0.55%)	190 (0.22%)	2.53	1.25–4.54

**Table 3:** Detailed odds ratios for developing oral clefts.

	Case Mothers	Matched Controls	Comparison		
Type of CAs	No.	No.	Odds Ratio	95%–CI	
Oral clefts	110 (6.01%)	2393 (2.73%)	2.28	1.87–2.78	
Cleft palate with unilateral cleft lip	41 (2.24%)	920 (1.05%)	2.16	1.55–2.93	
Cleft lip	33 (1.80%)	649 (0.74%)	2.47	1.70–3.45	
Cleft palate, unspecified	32 (1.75%)	722 (0.82%)	2.15	1.47–3.01	

**Table 4:** Detailed odds ratios for developing congenital heart defects.

	Case Mothers	Matched Controls	Comparison	
Type of CAs	No.	No.	Odds Ratio	95%–CI
Cardiovascular malformations	190 (10.40%)	7296 (8.31%)	1.28	1.10–1.49
Ventricular septal defects	79 (4.32%)	2783 (3.17%)	1.38	1.09–1.72
Congenital malformations of heart, unspecified	29 (1.59%)	752 (0.86%)	1.86	1.25–2.66
Atrial septal defects	28 (1.53%)	1865 (2.13%)	0.72	0.48–1.02
Other congenital malformations of cardiac chambers and connections	12 (0.66%)	325 (0.37%)	1.78	0.94–3.02
Patent ductus arteriosus	12 (0.66%)	689 (0.79%)	0.83	0.44–1.41

**Table 5:** Detailed odds ratios for developing congenital abnormalities with or without folic acid supplementation.

	All		With Folic Acid	
Study Groups	No.	Odds Ratio, 95%–CI	No.	Odds Ratio, 95%–CI
Neural tube defects	88	2.22; (1.78–2.76)	39	2.20; (1.59–3.05)
Oral clefts	110	2.28; (1.87–2.78)	38	1.78; (1.28–2.48)
Congenital heart defects	190	1.28; (1.10–1.49)	68	0.90; (0.70–1.16)

**Table 6:** Detailed odds ratios for developing congenital abnormalities with or without maternal vitamin supplementation.

	All		With Maternal Vitamin Intake	
Study Groups	No.	Odds Ratio, 95%–CI	No.	Odds Ratio, 95%–CI
Neural tube defects	88	2.22; (1.78–2.76)	3	0.92; (0.29–2.88)
Oral clefts	110	2.28; (1.87–2.78)	10	2.73; (1.43–5.22)
Congenital heart defects	190	1.28; (1.10–1.49)	25	2.66; (1.72–4.12)

**Table 7:** Detailed odds ratios for developing congenital abnormalities with or without antipyretic drugs.

	All		With Antipyretic Drugs	
Study Groups	No.	Odds Ratio, 95%–CI	No.	Odds Ratio, 95%–CI
Neural tube defects	88	2.22; (1.78–2.76)	21	1.73; (1.12–2.69)
Oral clefts	110	2.28; (1.87–2.78)	35	2.43; (1.72–3.42)
Congenital heart defects	190	1.28; (1.10–1.49)	64	1.41; (1.08–1.83)

## Data Availability

All data are included in the manuscript.

## Supplementary Materials

The following supporting information can be downloaded at https://www.mdpi.com/article/10.3390/jcm12216934/s1: Table S1. The STROBE Checklist, Table S2. The prevalence of specific types of congenital anomalies after first-trimester influenza infection.

## References

[1] Morens D.M., Daszak P., Markel H., Taubenberger J.K. (2020). Pandemic COVID-19 Joins History’s Pandemic Legion. mBio.

[2] Pleschka S. (2013). Overview of influenza viruses. Current Topics in Microbiology and Immunology.

[3] Silasi M., Cardenas I., Kwon J.Y., Racicot K., Aldo P., Mor G. (2015). Viral infections during pregnancy. Am. J. Reprod. Immunol..

[4] Mai C.T., Isenburg J.L., Canfield M.A., Meyer R.E., Correa A., Alverson C.J., Lupo P.J., Riehle-Colarusso T., Cho S.J., Aggarwal D. (2019). National population-based estimates for major birth defects, 2010–2014. Birth Defects Res..

[5] Mátrai Á., Teutsch B., Váradi A., Hegyi P., Pethő B., Fujisawa A., Váncsa S., Lintner B., Melczer Z., Ács N. (2022). First-Trimester Influenza Infection Increases the Odds of Non-Chromosomal Birth Defects: A Systematic Review and Meta-Analysis. Viruses.

[6] von Elm E., Altman D.G., Egger M., Pocock S.J., Gøtzsche P.C., Vandenbroucke J.P. (2007). The Strengthening the Reporting of Observational Studies in Epidemiology (STROBE) statement: Guidelines for reporting observational studies. Ann. Intern. Med..

[7] Czeizel A.E., Rockenbauer M., Siffel C., Varga E. (2001). Description and mission evaluation of the Hungarian case-control surveillance of congenital abnormalities, 1980–1996. Teratology.

[8] Ács N., Mátrai Á., Kaposi A. (2021). First data from the new, unified database of the Hungarian case-control surveillance of congenital abnormalities. J. Matern.-Fetal Neonatal Med..

[9] Czeizel A.E., Métneki J., Béres J. (2014). 50 years of the Hungarian Congenital Abnormality Registry. Congenit. Anom..

[10] R Core Team (2018). A Language and Environment for Statistical Computing.

[11] Czeizel A.E. (2008). Specified critical period of different congenital abnormalities: A new approach for human teratological studies. Congenit. Anom..

[12] Acs N., Bánhidy F., Puhó E., Czeizel A.E. (2005). Maternal influenza during pregnancy and risk of congenital abnormalities in offspring. Birth Defects Res. A Clin. Mol. Teratol..

[13] Li M., Liu Z., Lin Y., Chen X., Li S., You F., Deng Y., Li N., Wang Y., Zhang Y. (2014). Maternal influenza-like illness, medication use during pregnancy and risk of congenital heart defects in offspring. J. Matern.-Fetal Neonatal Med..

[14] Botto L.D., Lynberg M.C., Erickson J.D. (2001). Congenital heart defects, maternal febrile illness, and multivitamin use: A population-based study. Epidemiology.

[15] Oster M.E., Riehle-Colarusso T., Alverson C.J., Correa A. (2011). Associations between maternal fever and influenza and congenital heart defects. J. Pediatr..

[16] Saxén L., Holmberg P.C., Kurppa K., Kuosma E., Pyhälä R. (1990). Influenza epidemics and anencephaly. Am. J. Public Health.

[17] Kurppa K., Holmberg P.C., Kuosma E., Aro T., Saxén L. (1991). Anencephaly and maternal common cold. Teratology.

[18] Avagliano L., Massa V., George T.M., Qureshy S., Bulfamante G.P., Finnell R.H. (2019). Overview on neural tube defects: From development to physical characteristics. Birth Defects Res..

[19] Allam E., Windsor L., Stone C. (2014). Cleft lip and palate: Etiology, epidemiology, preventive and intervention strategies. Anat. Physiol..

[20] Saxén I. (1975). The association between maternal influenza, drug consumption and oral clefts. Acta Odontol. Scand..

[21] Saxén I. (1975). Epidemiology of cleft lip and palate. An attempt to rule out chance correlations. Br. J. Prev. Soc. Med..

[22] Dymanus K., Chishom T., Moraczewski J., Carroll W., Lima M., Yu J.C., Linder D. (2021). The Association Between Influenza Infection Rates and the Incidence of Orofacial Clefts in the United States. FACE.

[23] Ács L., Bányai D., Nemes B., Nagy K., Ács N., Bánhidy F., Rózsa N. (2020). Maternal-related factors in the origin of isolated cleft palate—A population-based case-control study. Orthod. Craniofacial Res..

[24] Voigt A., Radlanski R.J., Sarioglu N., Schmidt G. (2017). Cleft lip and palate. Pathologe.

[25] Ye Z., Wang L., Yang T., Chen L., Wang T., Chen L., Zhao L., Zhang S., Zheng Z., Luo L. (2019). Maternal Viral Infection and Risk of Fetal Congenital Heart Diseases: A Meta-Analysis of Observational Studies. J. Am. Heart Assoc..

[26] Ou Y., Mai J., Zhuang J., Liu X., Wu Y., Gao X., Nie Z., Qu Y., Chen J., Kielb C. (2016). Risk factors of different congenital heart defects in Guangdong, China. Pediatr. Res..

[27] Hegyi P., Erőss B., Izbéki F., Párniczky A., Szentesi A. (2021). Accelerating the translational medicine cycle: The Academia Europaea pilot. Nat. Med..

