# Supplementary material for: Reducing the Risk of Birth Defects Associated with Maternal Influenza: Insights from a Hungarian Case—Control Study
Source: J Clin Med. 2023 Nov 5;12(21):6934. doi: 10.3390/jcm12216934 (PMC10650197; doi:10.3390/jcm12216934)
Supplement: Supplementary file 1 [file jcm-12-06934-s001.zip › jcm-2665124-supplementary.pdf]

Reducing the Risk of Birth Defects Associated with Maternal Influenza: Insights from a Hungarian Case–Control Study

Authors

Ákos Mátrai[1,2,3], Brigitta Teutsch[3,4], Boglárka Pethő[1,3], András D. Kaposi[5], Péter Hegyi[3,4,6], Nándor Ács[1]

Affiliations:

1. Department of Obstetrics and Gynecology, Semmelweis University, 1082 Budapest, Hungary
2. Faculty of Health Sciences, University of Pécs, 7621 Pécs, Hungary
3. Centre for Translational Medicine, Semmelweis University, 1085 Budapest, Hungary
4. Institute for Translational Medicine, Medical School, University of Pécs, 7623 Pécs, Hungary
5. Department of Biophysics and Radiation Biology, Semmelweis University School of Medicine, 1094 Budapest, Hungary
6. Institute of Pancreatic Diseases, Semmelweis University, 1083 Budapest, Hungary

Corresponding author

Nándor Ács MD, PhD

Head of Department
Department of Obstetrics and Gynecology
Semmelweis University School of Medicine
Postal address: HU-1082, Budapest, Üllői út 78/A

Tel.: +36309520861

E-mail address: acsnandor@gmail.com

Running title:

First trimester maternal influenza and non-chromosomal birth defects

FIGURE LEGENDS

Table S1._ representing the STROBE Checklist

Table S2. representing the prevalence of specific types of congenital anomalies after first-trimester influenza infection.

Table S1._ representing the STROBE Checklist

	Item No.	Recommendation	Page No.
**Title and abstract**	1	(*a*) Indicate the study's design with a commonly used term in the title or the abstract	1
		(*b*) Provide in the abstract an informative and balanced summary of what was done and what was found	1
**Introduction**			
Background/rationale	2	Explain the scientific background and rationale for the investigation being reported	1
Objectives	3	State specific objectives, including any prespecified hypotheses	2
**Methods**			
Study design	4	Present key elements of study design early in the paper	2
Setting	5	Describe the setting, locations, and relevant dates, including periods of recruitment, exposure, follow-up, and data collection	2
Participants	6	(*a*) *Cohort study*—Give the eligibility criteria, and the sources and methods of selection of participants. Describe methods of follow-up<br>*Case-control study*—Give the eligibility criteria, and the sources and methods of case ascertainment and control selection. Give the rationale for the choice of cases and controls<br>*Cross-sectional study*—Give the eligibility criteria, and the sources and methods of selection of participants	2,3

		(*b*) *Cohort study*—For matched studies, give matching criteria and number of exposed and unexposed<br>*Case-control study*—For matched studies, give matching criteria and the number of controls per case	2,3
Variables	7	Clearly define all outcomes, exposures, predictors, potential confounders, and effect modifiers. Give diagnostic criteria, if applicable	3
Data sources/ measurement	8*	For each variable of interest, give sources of data and details of methods of assessment (measurement). Describe comparability of assessment methods if there is more than one group	3,4
Bias	9	Describe any efforts to address potential sources of bias	9
Study size	10	Explain how the study size was arrived at	4

Continued on next page

Quantitative variables	11	Explain how quantitative variables were handled in the analyses. If applicable, describe which groupings were chosen and why	4
Statistical methods	12	(*a*) Describe all statistical methods, including those used to control for confounding	4
		(*b*) Describe any methods used to examine subgroups and interactions	4
		(*c*) Explain how missing data were addressed	4
		(*d*) *Cohort study*—If applicable, explain how loss to follow-up was addressed<br>*Case-control study*—If applicable, explain how matching of cases and controls was addressed<br>*Cross-sectional study*—If applicable, describe analytical methods taking account of sampling strategy	4
		(*e*) Describe any sensitivity analyses	4
**Results**			
Participants	13*	(a) Report numbers of individuals at each stage of study—eg numbers potentially eligible, examined for eligibility, confirmed eligible, included in the study, completing follow-up, and analysed	4,5
		(b) Give reasons for non-participation at each stage	4,5
		(c) Consider use of a flow diagram	5
Descriptive data	14*	(a) Give characteristics of study participants (eg demographic, clinical, social) and information on exposures and potential confounders	5
		(b) Indicate number of participants with missing data for each variable of interest	5
		(c) *Cohort study*—Summarise follow-up time (eg, average and total amount)	
Outcome data	15*	*Cohort study*—Report numbers of outcome events or summary measures over time	
		Case-control study—Report numbers in each exposure category, or summary measures of exposure	*5,6*
		Cross-sectional study—Report numbers of outcome events or summary measures	
Main results	16	(*a*) Give unadjusted estimates and, if applicable, confounder-adjusted estimates and their precision (eg, 95% confidence interval). Make clear which confounders were adjusted for and why they were included	6
		(*b*) Report category boundaries when continuous variables were categorized	6,7
		(*c*) If relevant, consider translating estimates of relative risk into absolute risk for a meaningful time period	

Continued on next page

Other analyses	17	Report other analyses done—eg analyses of subgroups and interactions, and sensitivity analyses	7
**Discussion**			
Key results	18	Summarise key results with reference to study objectives	8
Limitations	19	Discuss limitations of the study, taking into account sources of potential bias or imprecision. Discuss both direction and magnitude of any potential bias	9
Interpretation	20	Give a cautious overall interpretation of results considering objectives, limitations, multiplicity of analyses, results from similar studies, and other relevant evidence	9
Generalisability	21	Discuss the generalisability (external validity) of the study results	9,10
**Other information**			
Funding	22	Give the source of funding and the role of the funders for the present study and, if applicable, for the original study on which the present article is based	10

*Give information separately for cases and controls in case-control studies and, if applicable, for exposed and unexposed groups in cohort and cross-sectional studies.

Note: An Explanation and Elaboration article discusses each checklist item and gives methodological background and published examples of transparent reporting. The STROBE checklist is best used in conjunction with this article (freely available on the Web sites of PLoS Medicine at http://www.plosmedicine.org/, Annals of Internal Medicine at http://www.annals.org/, and Epidemiology at http://www.epidem.com/). Information on the STROBE Initiative is available at www.strobe-statement.org.

Table S2. representing the prevalence of specific types of congenital anomalies after first- trimester influenza infection

ICD-10	Congenital malformation	No.
Q0000	Anencephaly	79
Q0010	Craniorachischisis	69
Q0020	Iniencephaly	62
Q0100	Frontal encephalocele	41
Q0110	Frontal encephalocele	41
Q0120	Occipital encephalocele	33
Q0180	Encephalocele of other sites	32
Q0190	Encephalocele, unspecified	30
Q02H0	Microcephaly	28
Q0300	Malformations of aqueduct of Sylvius	19
Q0310	Atresia of foramina of Magendie and Luschka	19
Q0380	Other congenital hydrocephalus	19
Q0390	Congenital hydrocephalus, unspecified	19
Q0400	Congenital malformations of corpus callosum	14
Q0410	Arhinencephaly	13
Q0420	Holoprosencephaly	13
Q0430	Other reduction deformities of brain	12
Q0440	Septo-optic dysplasia	12
Q0450	Megalencephaly	12
Q0460	Congenital cerebral cysts	11
Q0480	Other specified congenital malformations of brain	10
Q0490	Congenital malformation of brain, unspecified	10
Q0500	Cervical spina bifida with hydrocephalus	9
Q0510	Thoracic spina bifida with hydrocephalus	9
Q0520	Lumbar spina bifida with hydrocephalus	9
Q0530	Sacral spina bifida with hydrocephalus	9
Q0540	Unspecified spina bifida with hydrocephalus	8
Q0550	Cervical spina bifida without hydrocephalus	8
Q0560	Thoracic spina bifida without hydrocephalus	7
Q0570	Lumbar spina bifida without hydrocephalus	6
Q0580	Sacral spina bifida without hydrocephalus	6
Q0590	Spina bifida, unspecified	6
Q0600	Amyelia	6
Q0610	Hypoplasia and dysplasia of spinal cord	5
Q0620	Diastematomyelia	5
Q0630	Other congenital cauda equina malformations	5
Q0640	Hydromyelia	5

Q0680	Other specified congenital malformations of spinal cord	5
Q0690	Congenital malformation of spinal cord, unspecified	5
Q0700	Arnold-Chiari syndrome	4
Q0780	Other specified congenital malformations of nervous system	4
Q0790	Congenital malformation of nervous system, unspecified	4
Q1010	Congenital ectropion	4
Q1020	Congenital entropion	4
Q1030	Other congenital malformations of eyelid	3
Q1040	Absence and agenesis of lacrimal apparatus	3
Q1050	Congenital stenosis and stricture of lacrimal duct	3
Q1060	Other congenital malformations of lacrimal apparatus	3
Q1070	Congenital malformation of orbit	3
Q1100	Cystic eyeball	3
Q1110	Other anophthalmos	2
Q1120	Microphthalmos	2
Q1130	Macrophthalmos	2
Q1200	Congenital cataract	2
Q1210	Congenital displaced lens	2
Q1220	Coloboma of lens	2
Q1230	Congenital aphakia	2
Q1240	Spherophakia	2
Q1280	Other congenital lens malformations	2
Q1290	Congenital lens malformation, unspecified	2
Q1300	Coloboma of iris	2
Q1310	Absence of iris	2
Q1320	Other congenital malformations of iris	2
Q1330	Congenital corneal opacity	2
Q1340	Other congenital corneal malformations	2
Q1350	Blue sclera	2
Q1380	Other congenital malformations of anterior segment of eye	2
Q1390	Congenital malformation of anterior segment of eye, unspecified	1
Q1400	Congenital malformation of vitreous humour	1
Q1410	Congenital malformation of retina	1
Q1420	Congenital malformation of optic disc	1
Q1430	Congenital malformation of choroid	1
Q1480	Other congenital malformations of posterior segment of eye	1
Q1490	Congenital malformation of posterior segment of eye, unspecified	1
Q1500	Congenital glaucoma	1
Q1580	Other specified congenital malformations of eye	1
Q1590	Congenital malformation of eye, unspecified	1
Q1600	Congenital absence of (ear) auricle	1

Q1610	Congenital absence, atresia and stricture of auditory canal (external)	1
Q1620	Absence of eustachian tube	1
Q1630	Congenital malformation of ear ossicles	1
Q1640	Other congenital malformations of middle ear	1
Q1650	Congenital malformation of inner ear	1
Q1690	Congenital malformation of ear causing impairment of hearing, unspecified	1
Q1700	Accessory auricle	1
Q1710	Macrotia	1
Q1720	Microtia	1
Q1730	Other misshapen ear	1
Q1740	Misplaced ear	1
Q1750	Prominent ear	1
Q1780	Other specified congenital malformations of ear	1
Q1790	Congenital malformation of ear, unspecified	1
Q1800	Sinus, fistula and cyst of branchial cleft	1
Q1810	Preauricular sinus and cyst	1
Q1820	Other branchial cleft malformations	1
Q1830	Webbing of neck	1
Q1840	Macrostomia	1
Q1850	Microstomia	1
Q1860	Macrocheilia	1
Q1870	Microcheilia	1
Q1880	Other specified congenital malformations of face and neck	1
Q1890	Congenital malformation of face and neck, unspecified	1
Q2000	Common arterial trunk	1
Q2010	Double outlet right ventricle	1
Q2020	Double outlet left ventricle	1
Q2030	Discordant ventriculoarterial connection	1
Q2040	Double inlet ventricle	1
Q2050	Discordant atrioventricular connection	1
Q2060	Isomerism of atrial appendages	1
Q2080	Other congenital malformations of cardiac chambers and connections	1
Q2090	Congenital malformation of cardiac chambers and connections, unspecified	1
Q2100	Ventricular septal defect	1
Q2110	Atrial septal defect	1
Q2120	Atrioventricular septal defect	1
Q2130	Tetralogy of Fallot	1
Q2140	Aortopulmonary septal defect	1
Q2180	Other congenital malformations of cardiac septa	1
Q2190	Congenital malformation of cardiac septum, unspecified	1
Q2200	Pulmonary valve atresia	1

Q2210	Congenital pulmonary valve stenosis	1
Q2220	Congenital pulmonary valve insufficiency	1
Q2230	Other congenital malformations of pulmonary valve	1
Q2240	Congenital tricuspid stenosis	1
Q2250	Ebstein's anomaly	1
Q2260	Hypoplastic right heart syndrome	1
Q2280	Other congenital malformations of tricuspid valve	1
